# Supplementary material for: Mechanisms governing the pioneering and redistribution capabilities of the non-classical pioneer PU.1
Source: Nat Commun. 2020 Jan 21;11:402. doi: 10.1038/s41467-019-13960-2 (PMC6972792; doi:10.1038/s41467-019-13960-2)
Supplement: Supplementary file 7 — Source data [file 41467_2019_13960_MOESM7_ESM.zip › Source_Data/FigureS2/FigureS2B_MotifScanOutput/KmeansCluster1/homerResults/motif27.similar.html]

motif27

## Information for motif27

G
A
C
T
G
A
C
T
T
G
C
A
G
C
A
T
C
G
T
A
A
G
T
C
G
T
A
C
G
A
C
T
C
T
G
A
C
A
T
G
C
T
G
A
T
C
G
A
T
C
G
A
  
Reverse Opposite:  

A
G
C
T
A
C
G
T
A
G
C
T
G
T
A
C
G
A
C
T
C
T
G
A
C
A
T
G
T
C
A
G
G
C
A
T
C
G
T
A
A
C
G
T
C
T
G
A
C
T
G
A
  

|  |  |
| --- | --- |
| p-value: | 1e-29 |
| log p-value: | -6.715e+01 |
| Information Content per bp: | 1.708 |
| Number of Target Sequences with motif | 105.0 |
| Percentage of Target Sequences with motif | 2.06% |
| Number of Background Sequences with motif | 176.9 |
| Percentage of Background Sequences with motif | 0.45% |
| Average Position of motif in Targets | 94.7 +/- 48.2bp |
| Average Position of motif in Background | 104.5 +/- 68.7bp |
| Strand Bias (log2 ratio + to - strand density) | -0.1 |
| Multiplicity (# of sites on avg that occur together) | 1.00 |
| Motif File: | file (matrix) reverse opposite |

### Similar de novo motifs found

|  |  |  |  |  |  |  |  |
| --- | --- | --- | --- | --- | --- | --- | --- |
| Rank | Match Score | Redundant Motif | P-value | log P-value | % of Targets | % of Background | Motif file |
| 1 | 0.889 | C G A T G C T A G C A T C T G A A T C G A G T C G A C T T C G A A T C G C T G A C T G A T C G A | 1e-24 | -57.398166 | 0.71% | 0.03% | motif file (matrix) |
| 2 | 0.918 | A C G T G T C A A C G T T C G A A G T C A T G C A C G T T C G A A T C G T C G A C T G A | 1e-24 | -55.870474 | 1.43% | 0.25% | motif file (matrix) |
| 3 | 0.872 | A C G T C G T A A G C T C G T A A G T C A T G C A C G T G T C A A C T G G T C A | 1e-16 | -38.803082 | 0.65% | 0.06% | motif file (matrix) |
| 4 | 0.814 | A C G T C G T A A G C T C G T A A G T C A T G C A C G T C G T A A T C G | 1e-15 | -35.936335 | 1.06% | 0.23% | motif file (matrix) |
